# Inequalities, the arts and public health: Towards an international conversation

**DOI:** 10.1080/17533015.2013.826260

**Published:** 2013-08-12

**Authors:** Clive Parkinson, Mike White

**Affiliations:** a Arts for Health, Manchester Metropolitan University, Manchester, UK; b Centre for Medical Humanities, School of Health and Medicine, Durham University, Durham, UK

**Keywords:** international, participatory arts, public health, inequalities, intrinsic/instrumental benefits

## Abstract

This paper considers how participatory arts informed by thinking in public health can play a significant part internationally in addressing inequalities in health. It looks beyond national overviews of arts and health to consider what would make for meaningful international practice, citing recent initiatives of national networks in English-speaking countries and examples of influential developments in South America and the European Union. In the context of public health thinking on inequalities and social justice, the paper posits what would make for good practice and appropriate research that impacts on policy. As the arts and health movement gathers momentum, the paper urges the arts to describe their potency in the policy-making arena in the most compelling ways to articulate their social, economic and cultural values. In the process, it identifies the reflexive consideration of participatory practice – involving people routinely marginalised from decision-making processes – as a possible avenue into this work.

## Background and Context

This paper considers the growing significance of addressing health inequalities in the international development of the arts and health field, and the positioning of this work in relation to public health and national cultural policies in different countries. It attempts to look beyond the various national pictures presented in previous issues of this journal and identify common ground for practice and research. Some concise examples of emergent arts and health policy and strategy are drawn from South America and some European Union member states.

In 2009, academics and practitioners involved in the field of arts and health contributed to a paper, entitled ‘The State of Arts and Health in England’ (Clift et al., 2009), which featured in the first issue of Arts & Health: An International Journal for Research, Policy and Practice. This new paper reappraises the field in light of the changing political landscape, casting its net wider to look beyond the English-speaking world presented in that England ‘state of the art’ (abbreviated as SOA) assessment and in subsequent SOA papers from the USA (Sonke, Rollins, Brandman, & Graham-Pole, 2009), Canada (Cox et al., 2010) and Australia (Wreford, 2010). In the process, the paper will neither restate the key points made in the original SOA papers nor relist organisations and contacts, however relevant they remain today. Rather, it seeks to open a dialogue with countries in which arts and health is an emergent practice and English is not spoken as a first language.

When the UK changed government in 2010, it seemed that the national arts and health agenda might already have run out of steam, just as an international picture was emerging in presentations at the annual conferences of the USA’s Society for Arts in Healthcare (www.sah.org) and Arts and Health Australia (www.artsandhealth.org). The UK’s National Network for Arts and Health (NNAH) collapsed in 2007, at a moment when practitioners and researchers needed to network with each other and engage in political advocacy at the highest level. Like many countries, the UK has been facing swingeing austerity measures to counter what has been termed a global economic crisis. The turmoil around cost-cutting and the vicious restructuring of the public sector has meant that the excitement which followed the publication of the Arts Council England and Department of Health’s (2007) prospectus for arts and health feels like something of a distant dream. Although the accompanying report of the Department of Health’s working group on arts and health (2007) declared positively that there was a wealth of good practice and a substantial evidence base and that the Department of Health had an important leadership role to play in creating an environment in which arts and health could prosper by promoting, developing and supporting arts and health, it appears in practical terms to have achieved little either in influencing funding policies or directly supporting practice in the field. As a consequence of this, the field has focused internally on its funding problems and has overlooked a changing international picture for arts and health that could align research interests around the relationship of culture, well-being and social justice, and offer opportunities for cross-national collaborations.

The impetus for an international field of arts and health came in 1978 when, under the auspices of the World Health Organisation (WHO), leaders in public health from both developed and developing nations met at Alma-Ata in the former Soviet Union to frame a declaration that the improved well-being of citizens was to be the millennial goal of global public health. From Alma-Ata forward, public health specialists began to think in terms of developing new social norms for health, empowering people towards personal growth and responsibility for their health actions, making increased use of the media for health education and building alliances and support systems which would enable individuals to make healthy choices. The Alma-Ata Declaration (WHO, 1978) led to the WHO’s (1981) *Strategy for Health for All* in which the strength and nature of local culture was seen as an important indicator of health (Hancock, 1983). It seemed the more local and specific that the interpretation of *Health for All* goals became, the more that cultural activity emerged as a key factor in their realisation. Attention by public health to what constitutes a healthy city,for example, has been paralleled in the cultural policy sphere by analysis of factors that drive the strategic development of the “creative city” (Landry, 2000). Common to both is a concern to support social inclusion by fostering meaning, identity and a sense of place through cultural engagement.

As was previously reported in this journal (Clift, Camic, & Daykin, 2010), the UK arts and health field has been unable to harness momentum for international interventions from the WHO report of the Commission on Social Determinants of Health (chaired by Sir Michael Marmot) *Closing the Gap in a Generation: Health Equity through Action on the Social Determinants of Health* (WHO, 2008). On the subject of measurement, the report says governments need to see the social gradient of health and recognise that “Evidence is only one part of what swings policy decisions – political will and institutional capacity are important too.” Inter-sectoral coherence both in planning and interdisciplinary research are also vital to addressing the social determinants of health, as the report states: “More than simply academic exercises, research is needed to generate new understanding in practical, accessible ways, recognising and utilising a range of types of evidence, and recognising the added value of globally expanded Knowledge Networks and communities.” The report argues that global action is essential to make a difference and is necessary as “social injustice is killing people on a grand scale.” Growing an international exchange of research and practice in arts and health could offer practical examples of what those “globally expanded Knowledge Networks and communities” might look like.

Following on from the *Closing the gap* report, in the Rio Political Declaration on Social Determinants of Health, the WHO describes the social determinants of health as the conditions in which people are born, grow, live, work and age, noting that these circumstances are shaped by the distribution of money, power and resources at global, national and local levels (WHO, 2011a, b). The WHO urges the commitment of its member states to take action in a number of priority areas, including promoting participation in policy-making and reorientating the health sector towards promoting health and reducing health inequities. The WHO talks about a need to engage with actors outside government. This includes civil society and, arguably, the arts and cultural sectors. So, this paper argues that creativity, the arts and culture might have a significant role in addressing unfair and avoidable differences in health status. Relatively invisible from the perspective of the WHO’s high-end policy-making, the arts will need to describe their potency in this arena in the most compelling ways. One avenue into this will be to consider participatory practice involving people routinely marginalised from decision-making processes by having the least access to the policy-making machinery. Drawn from practice in the field and emerging need, the considerations outlined here suggest that some common approaches might have been developing across national boundaries.

## The Arts, Public Health and Well-being

When Charles-Edward Winslow defined public health in 1920, he referred to the “science and art” of preventing disease, of education, of social machinery and the realisation of birth rights based on principles of equality – areas that require knowledge, imagination and political advocacy. Over the past century, public health has adapted to a gamut of health and social crises. Some of these overlap with those exposed by the early public health pioneers, not least the long pursuit of democracy and the need for tools to shape individual and social life. Other issues could only arise in unequal contemporary societies, such as obesity, addictive behaviours and emergent forms of zoonoses (animal diseases that are transmissible to humans).

With the growth of international collaborations in arts and health and an interdisciplinary research approach forged in the emergent field of medical humanities, this paper urges both practitioners and researchers to take a leap of faith from Winslow’s (1920) definition of public health to think about participatory arts and the public health agenda in terms of our current economic climate. In taking a historical perspective upon recent developments, two essays are particularly useful – one by Lang and Rayner (2012) in the *British Medical Journal* and the other by Hanlon, Carlisle, Hannah, Lyon, and Reilly (2012) in *Perspectives in Public Health Journal* – both of which tie into a shared agenda of re-imagining public health.

Hanlon et al. (2012) suggest that the influence of science has expanded enormously, reaping huge benefits across many domains of life, not least medicine. They argue, however, that:

faith in science has morphed into an ideology best called “scientism”. Under scientism, what really matter is that which is known empirically, can be supported by evidence, can be counted or measured and, above all, can be shown to be value for money. Concerns about evidence and value for money are important, but can cause problems when taken too far. (p. 237)

This is particularly the case, they note, if metrics are used as the sole means of determining success. These same authors comment that modernity itself is in decline “because its methods and mindset are increasingly recognised as unsustainable” (p. 235). According to this logic, some profound changes will be needed in order for us to learn our way into the future, and those interested in public health “will need to develop new ways of thinking, being and doing […] and new forms of understanding the world” (p. 235). In pushing for a broader understanding of diverse world views, they posit that we have become:

increasingly separated from nature and this has led to the ecological crisis. We are separated from each other, and that creates conflict and damaging forms of individualism and selfishness. We are separated from ourselves in that we tend not to be in touch with our own sense of purpose. Finally, the materialistic, objectivist conception of our world and ourselves is robbing us of a subjective connection with what might best be called “spirit”. (Hanlon et al., 2012, p. 238)

In much the same way, Lang and Rayner (2012) suggest that “the pursuit of health and progress have become tangled up with consumerism as though there are no environmental consequences for health” (p. 2). As an antidote, they rhetorically ask how it is possible to “reframe thinking about mental health, social exclusion, and inequalities in health,” without placing democracy at the heart of our thinking, which is understood as people having “a sense of – and actual engagement in – shaping society and life, particularly when we live in a world in which so many people are excluded from control or who experience a sense of alienation in their lives” (p. 2).

These two papers illuminate the current trend for attributing blame to individual behaviour, suggesting that, by focusing on the micro, we fail to see the bigger picture – the shaping forces that frame the way we live our lives. The authors cited above propose that our capability to think and plan on a large scale has been delegated to corporations, world elites and the dehumanised forces of the market, as though the process itself was not initiated by vested interests which too rarely consider health impacts. Both these papers indicate that public health seems to have lost any sense of vision, and call for the development of knowledge as a continual intellectual engagement that moves beyond data collection towards the open pursuit of social values, highlighting the role of interest groups and debate across society rather than within restricted scientific circles. This opens a window of opportunity for artists, educationalists, health and social reformers and policy-makers concerned with public health, who believe that addressing inequality is central to human well-being and who have the vision and tenacity to think differently, going beyond simplistic assumptions of “globalisation” in both its malign and neoliberal manifestations.

In “Against the Organisation of Misery?” Pickett and Dorling (2010) critique the influential Marmot Review, also known as *Fair society, healthy lives* (Marmot, 2010), positing that this research simply restates inequalities that were exposed in the early 1980s, to note that:

What is missing is the political courage to deal with the root causes of those social determinants. Why people smoke, rather than trying to get them to stop. Why people eat too much, commit violence, trust each other less, invest more money in their children’s education; rather than trying to understand the social inequalities that stand in their way. (p. 1232)

Their criticism is valid in that it points to the need to tackle inequalities rather than just drawing attention to them, but it overlooks the report’s call for restoring “imagination in the NHS,” and the clear pointers to action given in some of the policy directives, such as “Create and develop healthy and sustainable places and communities” and “Enable all children, young people and adults to maximise their capabilities and have control over their lives”, with a priority objective to “ensure that schools, families and communities work in partnership to reduce the gradient in health, well-being and resilience” (Marmot, 2010, p. 9). It is in such a grass-roots self-help approach that common ground may be found in international arts and health practice.

## Conversations in the Gods and Grass-Roots Networks

Throughout the development of the arts and health movement, there have been luminaries and champions – people whose vision and belief is not tempered by the fluctuating economy, who see the potential of the arts beyond artificial domains. In the UK, no one better illustrates this type of political advocacy than Lord Alan Howarth of Newport, who has embraced the arts as a vehicle for health and social change. Following publication of the Prospectus, he initiated an arts/health debate in the House of Lords, and compelled the last Labour Government’s Secretary of State for Health, Alan Johnson, MP, to deliver a speech in which he commended work within the field (Johnson, 2008). He is currently engaged in dialogue with politicians in a bid to further the arts and health agenda through the establishment of an All-Party Parliamentary Group on Arts and Health.

Other figures recognised to have played a considerable part in nurturing the movement include Sir Kenneth Calman, who, as Chief Medical Officer in the 1990s, convened the influential Windsor conference with the Nuffield Trust (Phillipp, 2002) and went on to establish the Centre for Medical Humanities at Durham. Similarly, Dr Richard Smith (2002) – who used his platform as editor of the *British Medical Journal* to contend that “diverting 0.5% of the healthcare budget to the arts would improve the health of people in Britain” (p. 1432) – introduced a connection between health, adaptation and acceptance.

Alongside such individual champions, the field has benefited from an array of professional networks. Since the demise of NNAH, the North West Arts and Health Network (NWAHN), centred on the north-west of England, has been typical of the informal regional and sub-regional networks that have emerged across the UK, which provide web-based platforms and events for disseminating details of training, funding and employment opportunities. Through facilitated grass-roots events, over 1000 members of the NWAHN contributed to a Manifesto for Arts, Health and Wellbeing (2011), which continues to act as an advocacy document for the field. Contributing to the second iteration of the Manifesto (Arts for Health, 2011-2012), Lord Howarth astutely advocated the place of culture and the arts in public policy:

What is at issue is the right each one of us has to be human. To be human is to identify and liberate our own authentic and best nature. That quest will sometimes be private and sometimes be communal, and in the end the one merges into the other as we make the world we inhabit a better place. Trust, arduousness, risk, self-expression, shared work are means of moving towards individual and collective integrity. Teaching and companionship sustain us; orthodoxy and exploitation blight us. Politics should be predicated on these values. (p. 11)

Seeing a need for some national cohesion between emerging regional arts and health networks, the London Arts and Health Forum has brought together regional network champions as a National Alliance for Arts, Health and Wellbeing (England), the purpose of which will be to galvanise grass-roots engagement throughout the sector and develop the website at www.artshealthandwellbeing.org.uk as an information hub. Building on the Manifesto at stakeholder events across England, the regional partners have developed a *National charter for arts, health and wellbeing* (Jackson, 2012). When viewed alongside the emerging UK Arts and Health Research Network and the successful Culture Health & Wellbeing International Conference hosted in Bristol in 2013, the field in the UK seems to be flourishing, albeit in the face of widening inequalities, and looking to build international partnerships.

In his book *Arts Development in Community Health: A Social Tonic*, White (2009) describes the arts and health movement as “a small-scale global phenomenon”. In a similar vein, in summing up their Canadian SOA paper, “Tipping the iceberg?”, Cox et al. (2010) alludes to the “unstoppable momentum wherein new work will surface, new practitioners will emerge and new connections will be forged” (p. 120). It appears that, in the middle of a global recession, the arts and health agenda is coming into its own. Accordingly, this paper explores the idea of the field as a fast-expanding natural phenomenon. However, there is a danger that interest shown by policy-makers in a field like arts and health may be short-lived and evidence-obsessed, particularly if it takes place during a period of uncertainty. We run the risk of exploding, like a cultural supernova, and rapidly fading into obscurity.

In the USA, the subscription-based Society for Arts in Healthcare has perhaps prematurely rebranded itself as the Global Alliance for Arts & Health, to become the first organisation of this type to imply global reach. Whilst concerns around the impact of globalisation upon local identity and cultural distinctiveness are growing, this is an intriguing development. The Alliance’s collaboration with the international Arch of Arts in Health conference, staged in Israel in 2013, potentially adds fuel to an area of the world torn apart by inequalities, and pushes it towards an ethical cultural cliff.

Unlike the US model, Arts Health Network Canada and the National Alliance in England remain subscription-free, and whilst Australia currently lacks a formal national arts and health network, much of its field has grown out of the community arts movement, in which the key arts and disability organisation, Arts Access Australia, has been operating for over 40 years. At the same time, the annual international Art of Good Health and Wellbeing conference, organised by Arts and Health Australia, galvanises grass-roots activity and research. When taken alongside the emergence of a *National arts and health framework* in Australia (Institute for Creative Health, 2013), this suggests that Australia is building upon what appears to be a genuine commitment on the part of leading government ministers to develop innovative new ways of tackling inequalities. Similar high-level ministerial support is evident in the Welsh Assembly’s endorsement of the Arts Council of Wales’ (2009) national Arts, Health and Wellbeing Action Plan for Wales. In turn, this is not dissimilar to the French concordat established between the Ministry of Culture and Communication (2009) and the arts and health organisation Musique et Santé for projects such as *Culture and Health* which promotes art in hospitals.

Scrutiny of SOA research papers from England, Australia, the USA and Canada contributes to this story, reflecting a diversity of activity across the health and well-being spectrum. Common to all countries is an awareness that health and well-being are best promoted within the communities in which people live and work, and, whilst arts and health practice has, more often than not, emerged in clinical settings, hospitals and clinics account for just a proportion of this story.

## Beyond the English-Speaking World

In 2009, the first international Latin American arts and health forum took place in Peru, in which partners developed a visionary statement, entitled *The Lima declaration on arts, health and development* (Pan-American Health Organisation, 2009). Embracing the whole Latin American continent, this was underwritten by a driving vision of the arts as a powerful force for social change, addressing health and economic inequalities. This reflects some of the groundbreaking public health work that has been taking place under the auspices of the *Arts for Behaviour Change* programme run by Canyon Ranch Institute (2011) in the shanty settlements around Lima. Centred on health literacy and household hygiene, this has been exploring collective solutions to concrete problems through the innovative Theatre for Health initiative.

Similarly, in Brazil, the cultural group, AfroReggae, emerged following the police massacre of 21 people in the local community and was featured in the award-winning 2005 documentary film *Favela Rising*. The group has gone on to establish an international profile for its pioneering work in taking young people out of the drug/gang culture of Rio de Janeiro’s *favelas*, harnessing the ingenuity and creativity of communities to provide positive alternatives for young people. Reportedly the sixth richest economy in the world, Brazil will play host to the Olympic and Paralympic Games in 2016; yet inequalities are endemic in Brazilian society, and environmental issues, particularly deforestation of the Amazon, are of pressing concern. What place will the arts have in giving voice to under-represented communities and influencing change beyond the pyrotechnics of the opening ceremonies?

It may be possible to look at how, internationally, informal systems of social support are being evolved through cultural programmes. Wilkinson (2005) has emphasised the health benefits arising from a more cohesive society. He argues the quality of the social life of a community is one of the most powerful determinants of health and that this is related to the degree of income inequality. The sociologist Ray Pahl (2003) concurs, seeing that the quality of our social relationship in micro-social worlds is coming to be regarded as having a vital role in maintaining and achieving better health. These researchers, however, do not adequately explain the structure and processes of social support that can mitigate the effects of health inequalities, perhaps because looking at the cultural aspects of this is not in their domain. A political incentive for change is also required and it is useful to look at international examples addressing health inequalities through cultural interventions.

In 2009, the Republic of Lithuania staged its first international conference on arts and health as part of Vilnius’s stint as European Capital of Culture. Since then, Lithuania has made incremental developments within arts and health. Led by the Lithuanian Artists’ Association Gallery, which initiated a discussion in Parliament in 2009, the Prime Minister, Andrius Kubilius, went on to form an inter-ministerial working group to actively investigate opportunities for arts for health in Lithuania (Lithuanian Government, 2009). Also key to this cultural shift has been the inspirational support of the office of the popular President, Dalia Grybauskaitė, together with advocacy on the part of the British Council. Dalia Grybauskaitė was formerly Vice-Minister of Foreign Affairs and Minister of Finance, also European Commissioner for Financial Programming and the Budget from 2004 to 2009. This work has progressed with political stealth, embedding arts and health into national cultural policy. In 2011, The Guidelines of Alteration of Lithuanian Culture Policy were presented to parliament (Lithuanian Government, 2011). The ministries for Culture, Health and Social Care and Employment agreed to collaborate on the implementation of projects that have a positive impact on the integration of art in the spheres of health and social care, improving the quality of life. Considering its turbulent history, Lithuania is navigating new territories, and this stepped approach has displayed sophistication and integrity.

At a Europe-wide level, the United Nations Education, Scientific and Cultural Organisation (UNESCO) supports a European Network of Cultural Administration Training Centres (ENCATC). This takes the arts and health as a dedicated thematic area, which is currently being steered by colleagues in Finland – a country that has embraced art and culture as a fundamental human right which should be available to everyone throughout their life course. The five-year Finnish Art and Culture for Well-being programme outlines a manifesto and vision that not only suggests an achievable and succinct action plan but also offers a mechanism for delivering it.

Having conducted a review of the field in 2008, 17 Finnish organisations – including ministries of education, culture, social affairs, health, employment and the environment, regional and local authorities, arts councils, academic institutions and the private and voluntary sectors – began working together to deliver a plan that is funded in part by the public purse and partly by the private sector (Liikanen, 2010). Crucially, this influential group of organisations is chaired by the Assistant Director General of the National Institute for Health and Welfare and head of the Division of Health and Welfare Policies and Social and Health Economics. With no government elections until 2015, this represents an ongoing political commitment.

The Finnish group acknowledges something of a universal truth – that health, well-being, art and culture have traditionally been kept separate at a policy level, with limited coordination between the sectors, added to which the good practice that exists is often tied to short-term projects that are not shared in any systemic way. One of the most important elements of this work is that it starts with three basic assertions: health and well-being can be promoted by means of art and culture; art and culture can enhance inclusion at the individual, community and societal levels; and everybody should have the right to participate in, and enjoy, the cultural life of the community. With this in mind, the group is working to bring the political, administrative and structural together across three priority areas:

- culture as a means of promoting social inclusion, capacity building, networking and participation in daily life and living environments;
- art and culture as part of social welfare and health promotion;
- supporting well-being at work through art and culture.

This is a realistic and achievable arts and health manifesto, led by a senior figure within the health system, with the input of key ministerial departments.

It would seem, then, that when it comes to research, policy and practice, it might be interesting to follow Latin American, Lithuanian and Finnish developments. We can all learn from each other’s good practice but we may also benefit by collaborating on a commonly agreed research agenda for assessing the effectiveness of the arts in addressing health inequalities.

## Intrinsic vs. Instrumental

There needs to be close consultation with the arts sector on the development of the arts and health research agenda. Many artists working in health care settings express concern that their practice will be reduced by administrators and researchers to a functional product or process. When taken together with previous examples of art being used as a vehicle for propaganda by oppressive ideologies (including neoliberalism), this provokes the concern that art which is seen to be somehow working to the service of the state, and the inequalities it perpetuates, runs the risk of provoking cynicism. It would be easy to make the mistake of thinking that the arts and public health agenda *is* instrumentalism, necessarily reducing culture and the arts to being a subservient tool for social engineers, well-intentioned or otherwise.

A report from the Wallace Foundation in the USA, *Gifts of the Muse* – *Reframing the Debate about the Benefits of the Arts*, argues that the arts need to reinstate their intrinsic as well as instrumental benefits, and distinguish between private and public benefits. The report sees studies of health and social benefits as being limited so far in methodology and data. It notes a weakness in the attribution of benefits (because health benefits could be due to other effective causes than arts) and considers that the most important instrumental benefits require sustained involvement in the arts. It argues that intrinsic benefits are not solely private but include the creation of social bonds and communal meanings. The trouble with promoting “intrinsic benefits” (p. 5), however, is that we go back to arts for art’s sake arguments and still focus on the individual experience more than the communal. Furthermore, in research commissioned by Arts Council England, Belfiore and Bennett (2006) deplore how public debate of the value of arts has come to be “dominated by what might best be termed the cult of the measurable” (p. 5), and how it has become inseparable from funding issues. They argue that the tension between intrinsic and instrumental positions is a sterile dichotomy.

Knell and Taylor (2011), in the Royal Society for the Arts (RSA) pamphlet, *Arts Funding, Austerity and the Big Society: Remaking the Case for the Arts*, reject the dichotomy of intrinsic versus instrumental value to propose a new spectrum spanning artistic instrumentalism and public good instrumentalism. Knell and Taylor argue for a re-evaluation of the cultural bigotry ingrained at both ends of the spectrum, suggesting a different possibility in making a robust instrumental case for arts funding in terms that recognise what is different and special about artistic participation and appreciation.

At the same time, the RSA authors make a compelling case for methodologies, such as Contingent Value and Willingness to Pay estimates, which place the public at the heart of decision-making. Referring to what Matarasso (2010) has called “Distributed Culture,” Knell and Taylor suggest that the public should be enabled to commission art and cultural activity directly as this is a model in which local communities are given public money to invest in local cultural production, supporting a cultural programme of their design and choosing. The result might be that “cultural organisations large and small would be competing by tender to create vibrant cultural programmes for communities” (Knell & Taylor, 2011). It is envisaged that those involved in such projects would have a greater understanding of the value of the process and product. But there is a danger that such quasi-democratic strategies would perpetuate inequalities whilst replicating the deskilling that is going on in other areas of the public sector.

The report *Measuring the value of cullture* (DCMS, 2010) reinforces the argument that the cultural sector will need to use the tools and concepts of economics to fully state their benefits in the prevailing language of policy appraisal and evaluation, and it specifically recommends “contingent valuation” and “choice modelling,” with some interest in measures of subjective well-being that may become acceptable in the Treasury Green Book that determines spending decisions. The key problem, however, is that:

Narrative accounts of cultural value are especially important as they provide a framework for our understanding of cultural value, but fail to represent the benefits of culture in a manner that is commensurable with other calls on the public purse. Narrative accounts remind us of the need to make the case for culture in a variety of ways. Political decisions are not merely technocratic exercises in economic valuation, and nor should they be. However, without the data offered by economic valuation techniques the richness of the narratives of cultural value are likely to be less influential. (DCMS, 2010, p. 9)

What is clear is that those working in the arts and health field need to define themselves in appropriate and reflexive ways, paying close attention to artistic and public good instrumentalism and capturing social and economic value in terms that do not negate their integrity and vision. As Bakhshi (2010) usefully notes in his essay, ‘Beauty: value beyond measure?’, the authors of the (UK) Treasury’s Green Book guidelines for cost–benefit analysis “recommend that a range of techniques be used to elicit non-market values, even if these are subjective” (p. 7). The arts sector needs to make the best case possible for the participatory arts and public health agenda, but so far it has not had an intelligent enough debate about how it might deepen public understanding of the ways in which the arts can create social and economic value without falling into the reductionist language of the marketplace.

Arts Council England’s 10-year strategic framework (2010), *Great Art for Everyone*, might not explicitly prioritise arts/health, but its five long-term goals emphasise instrumental public good outcomes alongside artistic excellence, providing scope for the development of arts opportunities for people and places in areas of least engagement. Perhaps all arts organisations need to think of themselves as social condensers, in which people can connect socially as well as culturally. What is certain is that those charged with improving public health need to think more deeply about how they can re-imagine their engagement with people.

The arts and health research agenda is vast, as there is now a broad spectrum of practice and it is still innovative and curious. We must not stifle that emergent vision and potential by only seeking a proven evidence base that is narrowly defined through experimental and ‘control’ designs. There is a danger of reducing the whole arts and health field to being some kind of ancillary treatment in health care. To adapt an aphorism of Oscar Wilde, there seems little point in knowing the price of arts interventions in a medical model of health care if we do not also appreciate their value in addressing inequalities through a social model of preventive health – which is where so much of the potential of arts and health lies in the international arena. The emergence of small cross-national collaborations brings a renewed significance to narrative-based research because of the need to respect and reconcile differing cultural nuances in the application of creativity to health promotion. The “healthy living” stories we generate and exchange are the very basis for international practice in arts in community health.

## Conclusions

In more effectively and critically beginning to articulate what the field does, possibilities are created for influencing policy and wider public understanding. Those active in the field need to consider whether they are ready to articulate their vision and muster their resources. By the same token, artists need to critically engage with the big issues of the day – ageing populations, social isolation, addictive behaviours, substance abuse, obesity and mental ill health – all of which are underpinned by inequality. Can we increase access to the arts without resorting to models that perpetuate inequalities, and can we redefine the social value of the arts without slavishly bowing to scientism?

For an emerging participatory arts and public health movement to flourish and reach beyond its own boundaries, it must embrace cultural activity that sits outside its own community of interest and advocate for the arts within wider policy contexts. By moving away from a focus on the sick individual towards healthy communal and civic engagement, we open up new ways of learning ourselves into the future, contributing to new and evolving collaborations that take into account our pasts, but imagine new and different futures. Those interested in public health and well-being need to form alliances, from the local to the international, learning from and evolving with those working in non-English-speaking countries. We need to flesh out the case for integrating the arts and culture into this movement, as agents for change working beyond our local boundaries and embracing diversity. Lang and Rayner (2012) tell us that:

Public health success is as much about imagination as evidence: challenging what is accepted as the so called normal … public health must regain the capacity and will to address complexity and dare to confront power. (p. 5)

The identification of agents of change is crucial too for the realisation of WHO’s Rio Declaration aim to engage with actors outside government. Culture and the arts are constantly in flux, both responding to and having an influence on the times. Whereas the Enlightenment led to significant leaps in science and the industrial revolution, in this millennium, globalisation, new technologies and the dream of democracy are having an unprecedented impact on civic voices – best evidenced by the Arab Spring. How will artists and those concerned with civil society and public health respond? As the moral philosopher P.R. Baelz (1979) observed: “To stress the importance of a vision of a healthy society is to set health in the context of culture and to relate it to human values” (p. 29). This values-based approach to health, coupled with Seedhouse’s (1980) assertion that health is “creative potential,” has been an important driver in the emergent international practice of arts in community health. For an alliance of the arts and public health movement to be more than a cultural supernova, however, it needs to be confident of its place in a wider cultural ecology that is both nuanced and critically connected.

The international arts and health conversation needs to be about extending knowledge, partnerships and awareness on a global scale, in a reciprocal way that allows for work around the social determinants of health to be culturally relevant, equitable and geographically specific, rather than a one-size-fits-all foreign policy. To embed culture and the arts within the vision, policy and practice of public health, we need to be confident about our offer. If we are to be part of the movement that has been slowly evolving from the grass roots, now is the time to really understand how we connect more deeply with public health and the endemic inequalities that underpin and threaten well-being.
